# Rare Presentation of Parsonage-Turner Syndrome Post Tdap Vaccination: A Case Report

**DOI:** 10.7759/cureus.113667

**Published:** 2026-07-30

**Authors:** Matthew Jervis, Anastasie Dunn-Pirio

**Affiliations:** 1 Medicine, California University of Science and Medicine, Colton, USA; 2 Neurosciences, University of California San Diego, San Diego, USA

**Keywords:** brachial neuritis, brachial plexopathy, case report, neurology, parsonage-turner syndrome, peripheral neuropathy

## Abstract

Parsonage-Turner Syndrome (PTS) is a rare disorder of the brachial plexus typically characterized by acute shoulder and/or upper extremity pain, followed by muscle weakness. Though the specific etiology of PTS is unclear, numerous associations are cited in the literature, including after routine vaccinations.

A previously healthy 22-year-old male developed subacute left upper extremity weakness and numbness < 24 hours following a routine Tdap (tetanus toxoid, reduced diphtheria toxoid, and acellular pertussis) booster vaccination, later evolving into diffuse weakness and sharp shoulder pain. Electromyography (EMG) findings were suggestive of early PTS, and subsequent magnetic resonance imaging (MRI) of the brachial plexus confirmed mild inflammatory changes consistent with brachial plexitis. Over several months, the patient showed clinical improvement with conservative management.

This case highlights a rare presentation of post-vaccination PTS, emphasizing the need for early recognition and supportive management. Although rare, clinicians should consider PTS in patients presenting with acute-onset upper extremity pain and weakness following vaccination.

## Introduction

Parsonage-Turner Syndrome (PTS), otherwise known as neuralgic amyotrophy, idiopathic brachial plexopathy, or acute brachial neuritis, is a rare disorder affecting the brachial plexus. PTS is traditionally characterized by acute, unilateral shoulder pain followed by varying degrees of upper extremity weakness and numbness. The incidence of PTS is estimated to be 1 to 3 per 100,000 individuals [1]. However, a 2015 study in the Netherlands found the local incidence to be 1 per 1,000, suggesting PTS may be underreported or underdiagnosed [2]. The condition has a male predilection, with a male-to-female ratio of 2:1, and the average age of onset is 40 years [1]. PTS is distinct from hereditary brachial plexus neuropathy (HBPN), a similarly presenting condition caused by a mutation in the SEPTIN-9 gene. While the exact etiology of PTS remains unknown, it is hypothesized to be immune-mediated [3,4]. Known associations include infection, surgery, rheumatic disease, trauma, immunizations, pregnancy and childbirth, and other miscellaneous conditions [5]. The two most commonly reported antecedent events are recent viral illness and recent immunization.

Diagnosing and treating PTS can be challenging, requiring a thorough physical examination and advanced diagnostic testing, such as electromyography (EMG), nerve conduction studies (NCS), and magnetic resonance imaging (MRI), to rule out other potential causes. Nearly half of all reported PTS patients present with no identifiable risk factors [5,6]. Treatment and recovery are highly variable, yet most cases spontaneously resolve with supportive care within months to years [7]. This case of a young, previously healthy male highlights the unpredictable nature of PTS and underscores the importance of maintaining a broad differential diagnosis when evaluating patients with acute musculoskeletal and neurological complaints.

## Case presentation

A previously healthy 22-year-old male college student developed subacute, left upper extremity weakness and numbness less than 24 hours after receiving a Tdap (tetanus toxoid, reduced diphtheria toxoid, and acellular pertussis) booster vaccination to his left shoulder. The patient had first noticed tingling in his left thumb. This progressed throughout the day to numbness in his left forearm, tingling in all his fingers, and weakened grip strength. He reported difficulty holding a pencil and bringing his first and second digits together. Although the pattern of motor weakness resembled anterior interosseous nerve syndrome, the concurrent sensory symptoms involving the thumb, forearm, and fingers were atypical for an isolated anterior interosseous neuropathy and suggested a more proximal lesion. Two to three days later, he developed sharp left shoulder pain after waking up, which was partially relieved with ibuprofen. At the time of the neurology consultation, which was 11 days post-vaccination, he reported pain that was shooting up the left side of his neck, though some of his other symptoms had improved. Specifically, he reported that his fingers felt normal except for his thumb, and his numbness and weakness had lessened as compared to the week of symptom onset.

Pertinent physical exam findings at the initial neurology consultation included left pronator drift, 4+/5 strength in the left deltoid, +1 DTRs R/L (deep tendon reflexes right/left), and sensation to light touch and pinprick decreased over the left forearm. Subsequent physical therapy evaluation was also remarkable for 4/5 strength in left shoulder flexion, 4/5 strength in left elbow flexion, and mild tenderness to supraspinatus insertion. The treating physician noted the unusual inverted sequence of events for PTS, with weakness and numbness preceding the onset of shoulder pain. Therefore, electromyography and a nerve conduction study were ordered.

**Figure 1:**
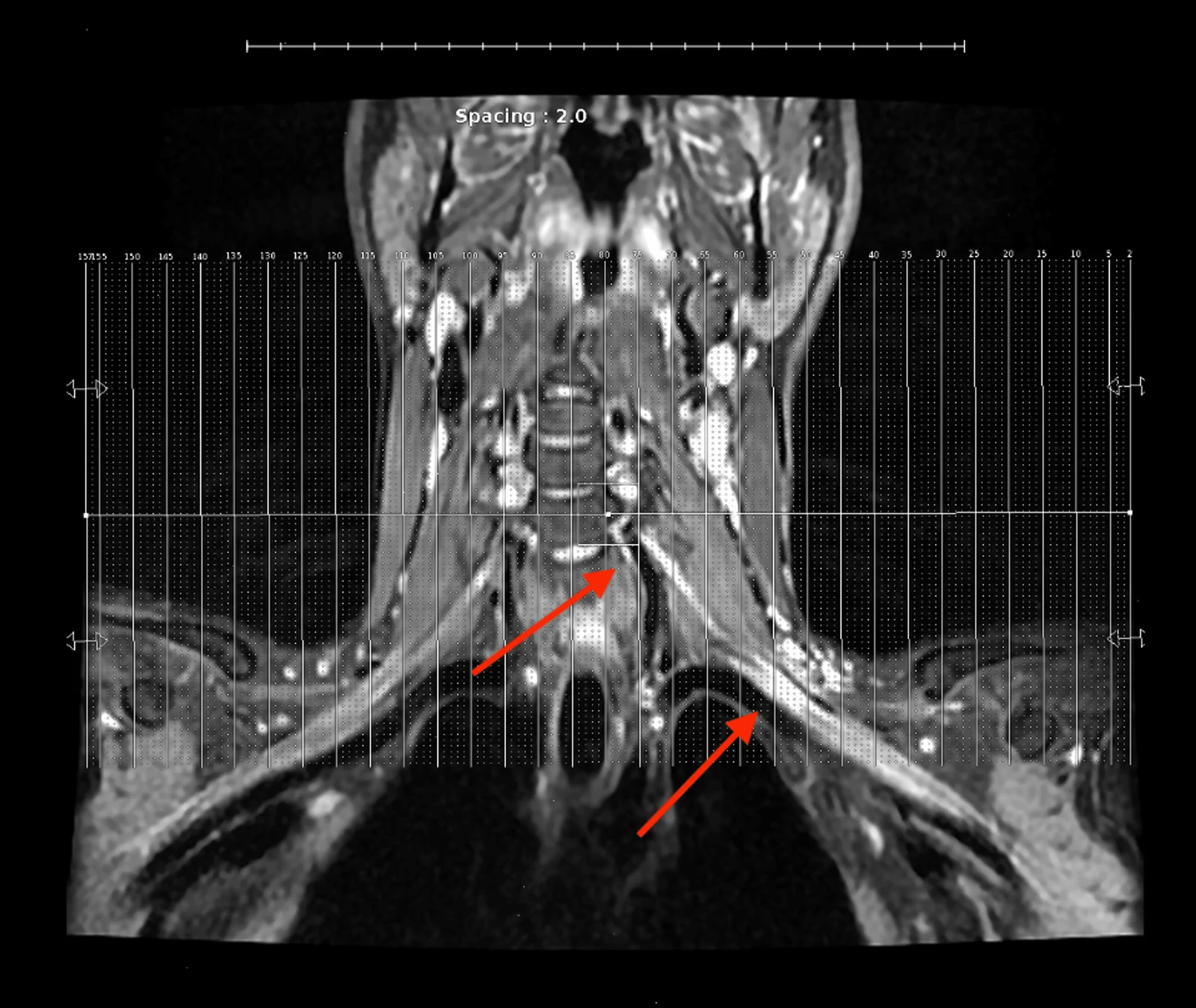
T2 cube coronal MRI L brachial plexus W/Wo contrast Enhancement, mild thickening, and nodularity of left brachial plexus, most prominently involving the left C5 and C6 nerve roots and left superior trunk.

**Figure 2:**
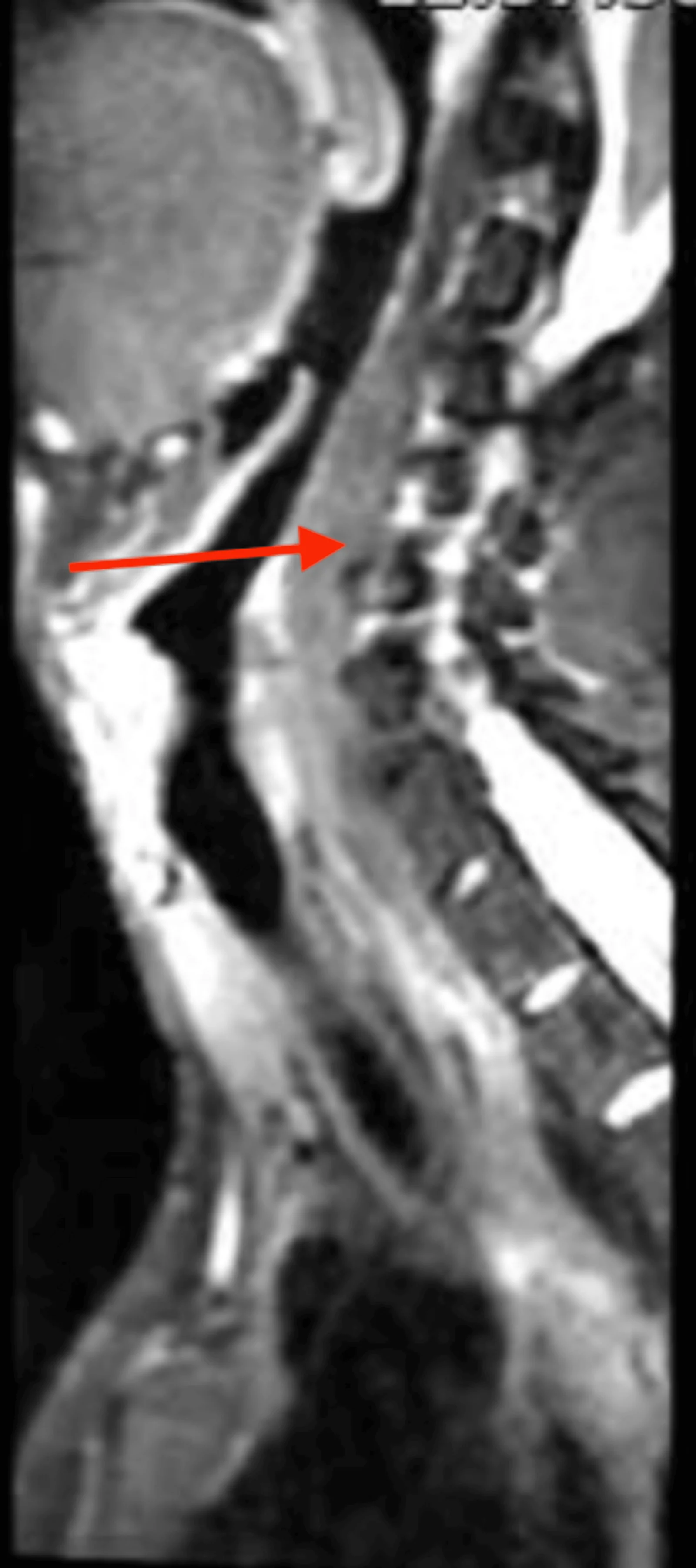
T2 cube sagittal MRI L brachial plexus W/Wo contrast Enhancement, mild thickening, and nodularity of left brachial plexus, most prominently involving the left C5 and C6 nerve roots and left superior trunk.

Initial EMG/NCS was performed 20 days post-vaccination. Needle EMG showed decreased recruitment in the left deltoid and biceps muscles, while sensory and motor nerve conduction studies were largely preserved. These electrodiagnostic findings, interpreted in conjunction with the clinical presentation, suggested involvement of the upper trunk of the brachial plexus rather than an isolated peripheral mononeuropathy. Because cervical radiculopathy remained in the differential diagnosis, MRI of the cervical spine without contrast was obtained and was unremarkable. MRI of the left brachial plexus with and without contrast demonstrated enhancement, mild thickening, and nodularity involving the left C5 and C6 nerve roots and superior trunk (Figures 1, 2), findings consistent with inflammatory brachial plexopathy. No mass was present in the adjacent soft tissues; there was prominence of the adjacent venous plexus at the base of the left neck, likely representing reactive hyperemia per the radiology report.

Repeat EMG/NCS was performed 3 months and 20 days post-vaccination while the patient’s symptoms were concurrently improving; these studies were normal. Tables 1-6 summarize the initial and repeat EMG/NCS findings. Upon final consultation with the patient (4.5 months post-vaccination), the patient reported significant overall improvement.

**Table 1:** Initial electromyography findings 20 days post-vaccination Abbreviations: IA, insertional activity; Fib, fibrillation potentials; PSW, positive sharp waves; Fasc, fasciculations; Amp, amplitude; Dur, duration; PPP, polyphasic potentials

	Spontaneous Activity					Voluntary Activity				Comment
Muscle	IA	Fib	PSW	Fasc	Misc	Amp	Dur	PPP	Recruitment	Pattern
L. Deltoid	Normal	None	None	None	None	Normal	Normal	Normal	Mild-Mod Decr	Neuropathic
L. Biceps brachii	Normal	None	None	None	None	Normal	Normal	Normal	Mild-Mod Decr	Neuropathic
L. Flexor carpi radialis	Normal	None	None	None	None	Normal	Normal	Normal	Normal	Normal
L. pronator teres	Normal	None	None	None	None	Normal	Normal	Normal	Normal	Normal
L. Triceps brachii	Normal	None	None	None	None	Normal	Normal	Normal	Normal	Normal
L. First dorsal interosseous	Normal	None	None	None	None	Normal	Normal	Normal	Normal	Normal
L. Rhomboid major	Normal	None	None	None	None	Normal	Normal	Normal	Normal	Normal
L. Serratus anterior	Normal	None	None	None	None	Normal	Normal	Normal	Normal	Normal

**Table 2:** Follow-up electromyography findings 3 months and 20 days post-vaccination Abbreviations: IA, insertional activity; Fib, fibrillation potentials; PSW, positive sharp waves; Fasc, fasciculations; Amp, amplitude; Dur, duration; PPP, polyphasic potentials

	Spontaneous Activity					Voluntary Activity				Comment
Muscle	IA	Fib	PSW	Fasc	Misc	Amp	Dur	PPP	Recruitment	Pattern
L. Deltoid	Normal	None	None	None	None	Normal	Normal	Normal	Normal	Normal
L. Biceps brachii	Normal	None	None	None	None	Normal	Normal	Normal	Normal	Normal
L. Flexor carpi radialis	Normal	None	None	None	None	Normal	Normal	Normal	Normal	Normal
L. pronator teres	Normal	None	None	None	None	Normal	Normal	Normal	Normal	Normal
L. Triceps brachii	Normal	None	None	None	None	Normal	Normal	Normal	Normal	Normal
L. First dorsal interosseous	Normal	None	None	None	None	Normal	Normal	Normal	Normal	Normal
L. Rhomboid major	Normal	None	None	None	None	Normal	Normal	Normal	Normal	Normal
L. Serratus anterior	Normal	None	None	None	None	Normal	Normal	Normal	Normal	Normal

**Table 3:** Initial sensory nerve conduction study findings 20 days post-vaccination Abbreviations: Rec., recording; Lat, latency; Amp, amplitude; ms, milliseconds; µV, microvolts; mm, millimeters; m/s, meters per second

Nerve / Site	Rec. Site	Onset Lat (ms)	Peak Lat (ms)	Amp (µV)	Segment	Distance (mm)	Velocity (m/s)
R Median – Digit II	Wrist	2.4	3.3	55	Wrist–II	140	57.4
L Median – Digit II	Wrist	2.6	3.5	46	Wrist–II	140	54.6
R Ulnar – Digit V	Wrist	2.4	3	31	Wrist–Digit V	120	50.5
L Ulnar – Digit V	Wrist	2.7	3.5	37	Wrist–Digit V	120	45
R Radial – Anatomical Snuff Box	Forearm	1.5	2.1	25	Forearm–Snuff Box	100	66.7
L Radial – Anatomical Snuff Box	Forearm	1.6	2.3	40	Forearm–Snuff Box	100	62.3
R Medial Antebrachial Cutaneous	Elbow	1.7	2.4	10	Elbow–Forearm	120	69.4
L Medial Antebrachial Cutaneous	Elbow	1.8	2.4	7	Elbow–Forearm	120	67
R Lateral Antebrachial Cutaneous	Elbow	1.7	2.2	22	Elbow–Forearm	120	72
L Lateral Antebrachial Cutaneous	Elbow	2	2.5	13	Elbow–Forearm	140	70.7

**Table 4:** Follow-up sensory nerve conduction study findings 3 months and 20 days post-vaccination Abbreviations: Rec., recording; Lat, latency; Amp, amplitude; ms, milliseconds; µV, microvolts; mm, millimeters; m/s, meters per second

Nerve / Site	Rec. Site	Onset Lat (ms)	Peak Lat (ms)	Amp (µV)	Segment	Distance (mm)	Velocity (m/s)
R Median – Digit II	Wrist	2.5	3.4	63	Wrist–II	140	55.5
L Median – Digit II	Wrist	2.5	3.4	57	Wrist–II	140	56.5
R Ulnar – Digit V	Wrist	2.1	3.1	45	Wrist–Digit V	120	55.9
L Ulnar – Digit V	Wrist	2.2	3	33	Wrist–Digit V	120	53.8
R Radial – Anatomical Snuff Box	Forearm	1.7	2.3	41	Forearm–Snuff Box	100	58.5
L Radial – Anatomical Snuff Box	Forearm	1.7	2.3	34	Forearm–Snuff Box	100	59.3
R Medial Antebrachial Cutaneous	Elbow	1.7	2.4	7	Elbow–Forearm	120	69.4
L Medial Antebrachial Cutaneous	Elbow	1.8	2.6	4	Elbow–Forearm	120	66.2
R Lateral Antebrachial Cutaneous	Elbow	1.8	2.4	14	Elbow–Forearm	120	65.5
L Lateral Antebrachial Cutaneous	Elbow	1.9	2.5	15	Elbow–Forearm	120	63.3

**Table 5:** Initial motor nerve conduction study findings 20 days post-vaccination Abbreviations: APB, abductor pollicis brevis; ADM, abductor digiti minimi; Rel. Amp, relative amplitude; Lat, latency; Amp, amplitude; ms, milliseconds; mV, millivolts; mV·ms, millivolt-milliseconds; mm, millimeters; m/s, meters per second

Nerve / Site	Muscle	Latency (ms)	Amplitude (mV)	Rel. Amp (%)	Duration (ms)	Area (mV·ms)	Segment	Distance (mm)	Lat Diff (ms)	Velocity (m/s)
R Median – Wrist	APB	3.3	12.3	100	5.1	28.2	Wrist–APB	70	—	—
R Median – Elbow	APB	7.8	9.1	74.5	5.4	22.4	Elbow–Wrist	260	4.5	57.2
L Median – Wrist	APB	3.4	13.5	100	5.6	38.1	Wrist–APB	70	—	—
L Median – Elbow	APB	8.1	13.5	99.6	5.6	38.1	Elbow–Wrist	270	4.6	58.1
R Ulnar – Wrist	ADM	2.4	9.9	100	5.4	26.8	Wrist–ADM	70	—	—
R Ulnar – Below Elbow	ADM	5.7	9.7	98.7	5.7	26.8	B.Elbow–Wrist	215	3.3	65.7
R Ulnar – Above Elbow	ADM	7.5	9.8	98.9	5.6	25.9	A.Elbow–B.Elbow	90	1.8	50.8
L Ulnar – Wrist	ADM	3.3	10.5	100	5.9	29	Wrist–ADM	70	—	—
L Ulnar – Below Elbow	ADM	7.1	9.2	87.2	7.6	30.9	B.Elbow–Wrist	220	3.8	58
L Ulnar – Above Elbow	ADM	9.1	8.6	81.8	7.4	31.8	A.Elbow–B.Elbow	100	2	49

**Table 6:** Follow-up motor nerve conduction study findings 3 months and 20 days post-vaccination Abbreviations: APB, abductor pollicis brevis; ADM, abductor digiti minimi; Rel. Amp, relative amplitude; Lat, latency; Amp, amplitude; ms, milliseconds; mV, millivolts; mV·ms, millivolt-milliseconds; mm, millimeters; m/s, meters per second

Nerve / Site	Muscle	Latency (ms)	Amplitude (mV)	Rel. Amp (%)	Duration (ms)	Area (mV·ms)	Segment	Distance (mm)	Lat Diff (ms)	Velocity (m/s)
R Median – Wrist	APB	3.5	9.4	100	5.9	31.2	Wrist–APB	70	—	—
R Median – Elbow	APB	7.8	8.7	92.2	7.5	29	Elbow–Wrist	245	4.3	57.6
L Median – Wrist	APB	2.9	8.8	100	5.5	27.6	Wrist–APB	70	—	—
L Median – Elbow	APB	7	8.7	99.5	5.7	26.5	Elbow–Wrist	240	4.1	58.8
R Ulnar – Wrist	ADM	2.5	17.4	100	5.5	51.7	Wrist–ADM	70	—	—
R Ulnar – Below Elbow	ADM	6.2	15.5	89.1	5.5	44.9	B.Elbow–Wrist	220	3.7	59
R Ulnar – Above Elbow	ADM	8.2	15.9	91.6	5.6	48.1	A.Elbow–B.Elbow	100	2	50.5
L Ulnar – Wrist	ADM	3	11.1	100	5.9	29.6	Wrist–ADM	70	—	—
L Ulnar – Below Elbow	ADM	6.4	10	90.9	6.1	27.8	B.Elbow–Wrist	200	3.4	59.6
L Ulnar – Above Elbow	ADM	8.8	8.9	80.8	6.2	27.6	A.Elbow–B.Elbow	100	2.4	41

## Discussion

Although PTS typically presents with acute unilateral shoulder pain followed by muscle weakness, this patient experienced these symptoms in reverse order. Additionally, symptom resolution occurred more rapidly than usual, with recovery lasting just over 4.5 months. Despite this atypical presentation, initial EMG/NCS findings were consistent with early PTS. MRI of the cervical spine was unremarkable, while MRI of the brachial plexus demonstrated mild inflammatory changes consistent with brachial plexopathy, further supporting the diagnosis of PTS [8].

The lack of other recognized antecedent events aside from recent vaccination, along with minor deviations from the traditional PTS presentation, highlights the importance of healthcare providers maintaining a high index of suspicion for PTS, even when symptoms deviate from the classic pattern. Clinicians should not prematurely rule out PTS based solely on an atypical symptom sequence and should pursue a thorough diagnostic evaluation when clinical suspicion remains.

While vaccination has been discussed in relation to PTS, a literature review revealed few published cases of PTS following Tdap vaccination [6,9,10]. A review of the Vaccine Adverse Event Reporting System (VAERS) revealed only 14 reports of “neuralgic amyotrophy," 12 reports of "brachial plexopathy," and 11 reports of "brachial plexitis" following Tdap vaccination across its more than three decades of data. Because VAERS is a passive surveillance system, these reports should be interpreted as hypothesis-generating safety signals and cannot be used to estimate the incidence or prevalence of PTS or establish causality. Nevertheless, the limited number of reports highlights an area warranting further investigation.

It is also noteworthy that the healthcare team collaborated effectively in this case. The primary treating physician promptly initiated orders for physical therapy and EMG/NCS testing. The physicians performing the initial EMG/NCS recommended repeat EMG/NCS testing in approximately three months to assess for interval electrodiagnostic changes and further characterize the extent of nerve injury.

## Conclusions

In summary, clinicians should consider PTS when developing differential diagnoses for patients presenting with acute musculoskeletal and neurological complaints, including those with a recent history of vaccination. This case demonstrates an atypical sequence of symptom onset alongside favorable recovery with conservative management, highlighting the importance of early recognition and appropriate diagnostic evaluation.
